# It’s time to transform the LGBT health research landscape in Malaysia

**DOI:** 10.1371/journal.pmen.0000110

**Published:** 2024-09-13

**Authors:** Kyle Tan, Jun Wei Liow

**Affiliations:** 1 Faculty of Māori and Indigenous Studies, University of Waikato, Hamilton, Aotearoa New Zealand; 2 Department of Social Work and Social Administration, The University of Hong Kong, Pokfulam, Hong Kong; PLOS: Public Library of Science, UNITED KINGDOM OF GREAT BRITAIN AND NORTHERN IRELAND

In 2024, at the time of writing this, Malaysia remains one of the 63 countries worldwide that criminalise lesbian, gay, bisexual, transgender and other queer (LGBT+) people based on their identities and expressions [1]. There is a penal code within secular law that punishes same-sex relations that the country inherited from the British colonial times in the 1800s. As a Muslim-majority country, there are also state-specific Sharia Islamic religious laws that persecute Muslim LGBT+ people. Collectively, these LGBT-criminalising laws engender an environment of fear for LGBT+ people–preventing them from affirming their identity, and creating fertile ground for explicit discrimination and violence against the communities and for an anti-LGBT+ rhetoric to flourish in the media and public [2]. LGBT+ people in Malaysia constitute an underserved population, and there is currently no law that prohibits discrimination based on sexual orientation, gender identity or expression, or sex characteristics.

The identity-criminalising context has direct implications for the conduct of LGBT+ physical and mental health research. Most notably, there are few to no local funding opportunities in Malaysia for LGBT health equity research. The few exceptions are when LGBT+ participants are included as part of research that is focused on vulnerable populations in a broader context [3], when research is funded by LGBT+ community organisations (e.g., Justice for Sisters) [4,5], or when studies are conducted as part of a postgraduate programme [6] as these generally fly under the radar of institutional scrutiny. Researchers may also be deterred by the potential repercussions of advocating for LGBT+ communities (e.g., legal persecution and online harassment), particularly when their institutions lack a track record of supporting LGBT+ equity. Further, we have experienced an infiltration of anti-LGBT rhetoric from the media into academic and research institutions [7], which attempts to be legitimised as empirical evidence and rarely intervened.

In this opinion, we seek to provide six pointers for expanding the capacity of culturally safe LGBT+ research in Malaysia that researchers can consider [8], as no such discussion currently exists. This is not intended to be an exhaustive list, and we encourage researchers to respond to these points according to their specific sociocultural contexts and the timely challenges faced by LGBT+ communities. Both authors are Malaysian scholars with experience conducting LGBT+ health research in Malaysia, and we acknowledge our privilege of being currently employed at overseas institutions that may shield us from specific anti-LGBT+ attacks.

1. *Liberatory research*. Often, a research project terminates after the extraction of data from LGBT+ communities, leaving them feeling disempowered and developing distrust towards researchers. The ‘publish or perish’ pressure in academia has led to a surge of lacklustre research that do not create social benefits. Prior to undertaking any LGBT+ health research, we encourage researchers to critically reflect on how their research can liberate LGBT+ communities from oppression and contribute to meaningful social change [9].

2. *Allies for colleagues and students*. While allies play crucial roles in creating platforms for LGBT+ health research, we must be mindful of our privilege (e.g., not having lived experience as a minoritised person due to cisgender and/or heterosexual identity) and engage in steps to redistribute power and resources to LGBT+ colleagues and students [10]. Some of these steps include being reflexive about own positionality, creating a pipeline for LGBT+ students in research, connecting students with LGBT+ community groups, and undertaking collaborative research [10].

3. *Prioritise community needs*. A recent scoping review found that most existing LGBT+ research in Malaysia focuses on topics related to sexually transmitted infections (STIs) [11]. It becomes apparent that there is a misalignment between researchers’ priorities and those of the community, as a community-based survey identified the removal of criminal laws and addressing related issues as top priorities for researchers, policy stakeholders, and governmental agencies [12]. As researchers, we have the power to shape the public narrative as ‘experts in the field’ and we can do justice to LGBT+ communities by generating data that challenge the status quo. We recommend that researchers in LGBT+ health work in interdisciplinary teams, including colleagues from public health, sociology, and community psychology, to examine the mechanisms underlying social determinants affecting LGBT people’s mental and behavioural health and to identify solutions to improve access to these determinants [13].

4. *Community-informed research*. Historically, LGBT+ communities have faced barriers to participating in research and influencing its direction, and there is a lack of research that celebrates community strengths. We recommend that researchers adopt a community-based participatory research approach to enhance academic-community partnerships [14]. Researchers can intentionally engage with LGBT+ community organisations or establish an advisory board comprising LGBT+ people with diverse representations (e.g., gender, ethnicity, and region) to participate in decision-making in all stages of the research. Given the phenomenon of over-research and consultation fatigue within LGBT+ communities, researchers ought to be mindful of not imposing additional cultural labour on LGBT+ community members and students (or colleagues) unless their role is adequately compensated.

5. *Cultural safety in ethics board*. Obtaining ethical approval for research projects on LGBT+ health in Malaysia can be challenging due to intense scrutiny, dismissal, or critiques from LGBT-phobic reviewers. This is especially difficult in public institutions, which may be reluctant to appear as endorsing LGBT+ equity. While we would like to see representation of LGBT+ members (including allies) on institutional ethics boards, this option is not always feasible, as many researchers may be unwilling to disclose their identities in homo/bi/transphobic environments. Therefore, we recommend at least including a cultural safety clause in ethics review protocols [8]. This clause should ensure that researchers reflect on their potential blind spots when working with minoritised communities, commit to doing no harm, and understand the social determinants of health affecting these communities [13].

6. *Innovative research*. There is an increasing number of studies producing similar results and recommendations that fail to address existing gaps in the literature [11]. Research is intended to generate new knowledge; however, the duplication of studies implies that LGBT+ participants may have contributed their perspectives to multiple projects on similar topics without advancing the field. We encourage researchers to conduct a thorough literature review, engage with LGBT+ community groups, and collaborate with researchers who have access to large datasets of LGBT+ participants (e.g., the KAMI Survey) [15] and experience in conducting community-led research [4,5].

At times, it may feel like a futile exercise when attempting to convince institutions and colleagues of the necessity for LGBT+ research that may not result in immediate changes, especially when faced with pushback, resistance, and disinformation. Nevertheless, as researchers, it is important to begin by identifying both local and international allies in the field, establishing connections with community groups, and building capacity for LGBT-affirmative research. By doing so, we can make progress toward gathering collective evidence to drive change at academic, community, and policy levels.

## References

[1] Human Dignity Trust. Map of jurisdictions that criminalise LGBT people. 2024 Jan 1 [Cited 2024 July 30]. Available from: https://www.humandignitytrust.org/lgbt-the-law/map-of-criminalisation/.

[2] Ting SH, Jerome C, Yeo J. Deviants or “normal” citizens?: Framing of LGBTQ in Malaysian newspapers. Asia Pac Soc Sci Rev. 2023;23: 14–28.

[3] Lim SH, Brown SE, Shaw SA, Kamarulzaman A, Altice FL, Beyrer C. “You have to keep yourself hidden”: Perspectives from Malaysian Malay-Muslim men who have sex with men on policy, network, community, and individual influences on HIV risk. J Homosex. 2020;67: 104–126. doi: 10.1080/00918369.2018.1525946 30307803

[4] Justice for Sisters, Diversity Malaysia, PLUHO, & Queer Lapis. Survey findings: Impact of Covid-19 & anti-LGBT narratives on LGBT persons in Malaysia. 2021 December 1 [Cited 2024 July 30]. Available from: https://www.queerlapis.com/survey-findings-impact-of-covid-19-anti-lgbt-narratives-on-lgbtq-persons-in-malaysia-out-now/.

[5] Justice for Sisters, Umbrella, L. U., PLUHO, & PLUsos. Survey: Conversion practices or efforts to change LGBTIQ+ people in Malaysia. 2023 April 1 [Cited 2024 July 30]. Available from: https://justiceforsisters.org/wp-content/uploads/2023/08/Conversion-Practice-Survey-Findings.pdf.

[6] Zay Hta MK, Tam CL, Au SY, Yeoh G, Tan MM, Lee ZY et al. Barriers and facilitators to professional mental health help-seeking behavior: Perspective of Malaysian LGBT individuals. J LGBT Issues Couns. 2021;15: 38–58. doi: 10.1080/15538605.2021.1868373

[7] Tan K. It’s time to consider LGBTQ-affirmative psychology in Malaysia. J Psychosex Health. 2022;4: 43–48. doi: 10.1177/26318318211060484

[8] Tan K, Ling SA. Cultural safety for LGBTQIA+ people: A narrative review and implications for health care in Malaysia. Sexes. 2022;3: 385–395. doi: 10.3390/sexes3030029

[9] Singh AA. Moving from affirmation to liberation in psychological practice with transgender and gender nonconforming clients. Am Psychol. 2016;71: 755–762. doi: 10.1037/amp0000106 27977258

[10] Streed CG, Perlson JE, Abrams MP, & Lett E. On, with, by—Advancing transgender health research and clinical practice. Health Equity. 2023;7(1):161–5. doi: 10.1089/heq.2022.0146 36895704 PMC9989508

[11] Tan K, Lee KW, Cheong ZW. Current research involving LGBTQ people in Malaysia: A scoping review informed by a health equity lens. J Popul Soc Stud. 2021;29: 622–643. doi: 10.25133/JPSSv292021.038

[12] Tan K. Setting the priorities for LGBT+ research and intervention effort in Malaysia through community voices: A brief report. Community Health Equity Res Policy. 2024; Forthcoming. doi: 10.1177/2752535X241273831 39120107 PMC12059226

[13] Ministry of Health. Health white paper for Malaysia: Strengthening people’s health, future-proofing the nation’s health system. 2023 June 15. [Cited 2024 July 30]. Available from: https://www.moh.gov.my/moh/resources/Penerbitan/Penerbitan%20Utama/Kertas%20Putih%20Kesihatan/Kertas_Putih_Kesihatan_(ENG)_compressed.pdf.

[14] Ricks JM, Arthur EK, Stryker SD, Yockey RA, Anderson AM, & Allensworth-Davies D. A systematic literature review of community-based participatory health research with sexual and gender minority communities. Health Equity. 2022;6: 640–657. doi: 10.1089/heq.2022.0039 36081887 PMC9448519

[15] Ho SH, Shamsudin AH, Liow JW, Juhari JA, Ling SA, Tan K. Mental healthcare needs and experiences of LGBT+ individuals in Malaysia: Utility, enablers, and barriers. Healthcare. 2024;12: 998. doi: 10.3390/healthcare12100998 38786409 PMC11120647

